# Electrophysiological Recordings from Embryonic Mouse Motoneurons Cultured on Electrospun Poly-Lactic Acid (PLA) and Polypyrrole-Coated PLA Scaffolds

**DOI:** 10.52547/ibj.26.3.183

**Published:** 2022-04-03

**Authors:** Esmeralda Zuñiga, Odin Ramírez, Carlos Martínez

**Affiliations:** 1 Avenida del Charro s/n y, Calle Henry Dunant, Omega, 32584 Cd Juárez, Chih. - Universidad Autonoma de Ciudad Juárez;; 2 Boulevard Calacoaya 7, La Ermita, 52970, Ciudad López Mateos, Mexico - Universidad Tecnologica de Mexico-UNITEC;; 3 Avenida del Charro s/n y, Calle Henry Dunant, Omega, 32584 Cd Juárez, Chih. - Universidad Autonoma de Ciudad Juárez

**Keywords:** Electrophysiology, Pyrrol, Scaffolds

## Abstract

**Background:**

Biomaterials used as cell growth stimulants should be able to provide adequate cell adhesion with no alteration in cell function. In this work, we developed a 3D model of mouse spinal cord motoneurons on scaffolds composed of electrospun PLA fibers and plasma-polymerized PPy-coated PLA fibers.

**Methods:**

The functionality of the cultured motoneurons was assessed by evaluating both the electrophysiological response (i.e., the whole-cell Na^+^ and K^+^ currents and the firing of action potentials) and also the expression of the VAChaT by immunostaining techniques. While the expression of the VAChaT was confirmed on motoneurons cultured on the fibrous scaffolds, the electrophysiological responses indicated Na^+^ and K^+^ currents with lower amplitude and slower action potentials when compared to the response recorded from spinal cord motoneurons cultured on Poly-DL-Ornithine/Laminin- and plasma-polymerized PPy-coated coverslips.

**Results:**

From a morphological viewpoint, motoneurons cultured on PLA and PPy-coated PLA scaffolds did not show the development of dendritic and/or axonal processes, which were satisfactorily observed in the bidimensional cultures.

**Conclusion:**

We hypothesize that the apparently limited development of dendritic and/or axonal processes could produce a deleterious effect on the electrophysiological response of the cells, which might be due to the limited growth surface available in the fibrous scaffolds and/or to an undesired effect of the purification process.

## INTRODUCTION

In tissues, cells are immersed in a 3D environment where certain biophysical, biochemical, and biomechanical characteristics influence key cellular functions such as migration, adhesion, proliferation, and gene xpression^[1-4]^. Owing to these features, the development of 3D scaffolds and cell culture models has gained increasing interest in recent years. Volumetric cultures of motoneurons and nerve cells from the spinal cord have been used as *in vitro *models to gain a better understanding of the pathophysiological mechanisms involved in nerve regeneration in spinal cord damage and neurodegenerative diseases and some other related pathologies^[5-9]^.

The 3D scaffolds are porous substrates designed to support cell growth, differentiation, and organization within and/or outside their structure. A great variety of 3D scaffolds have been proposed to comply the requirements of various cell types by providing these biomaterials with an optimal culturing environment in terms of morphology and chemical properties^[10,11]^. Considering factors such as biocompatibility, biodegradability, porosity and pore size, geometry, shape and size, and mechanical properties, 3D scaffolds have been developed using hydrogels, porous fibers, microspheres, and native tissue ^[11]^.

One of the key attributes of fibrous scaffolds is the porous structure having relatively large distances between the fibers, which allows and facilitates nutrition, gas exchange, and cell infiltration. Electrospinning is a technique broadly used to produce fibrous scaffolds. Using this technique, continuous fibers of diameters, ranging from nanometers to micrometers, can be produced, making these fibers suitable for numerous applications in the field of neural tissue engineering^[12-14]^. Among materials used to synthesize the fibrous scaffolds are collagen, hyaluronic acid, silk, chitosan, PLA, poly-glycolic acid/lactic acid, and poly-caprolactone^[15]^. A main advantage of electrospun fibrous scaffolds is that the orientation of the fibers, diameter, aspect ratio, surface area, and pore size can be controlled during the synthesis process^[12]^. The 3D scaffolds composed of electrospun fibers are commonly used to provide structural guidance for neurite outgrowth and axonal extension^[15]^. To potentiate cell growth and anchorage, both natural and synthetic polymers should be applied to modify the surface of both bidimensional and 3D scaffolds. One example of this is the use of plasma polymerization of pyrrole to provide surfaces with a crosslinked structure thin film that favors the interaction with cell surface integrins^[16-21]^, an effect associated with the presence of -NH and -OH groups and also other compounds such as 1H-azirine in the plasma PPy structure^[22-26]^.

In this work, we first cultured mouse embryo motoneurons on Poly-DL-Ornitina/Laminin- and Plasma PolyPPy-coated coverslips. Next, we developed a volumetric *in vitro *model of embryo motoneurons on PLA and PPy-coated PLA electrospun scaffolds. Finally, both cultures were evaluated functionally and morphologically by performing electrophysiological experiments using the whole-cell patch-clamp technique and immunostaining to confirm the presence of vesicular acetylcholine receptors.

## MATERIALS AND METHODS

**Electrospinning of PLA fibers**

The electrospinning setup consisted of a high voltage power supply (0-25 kV, Phywe, Germany), a homemade pumping system, a plastic syringe connected to a blunt tip 20G needle (0.6 mm inner radius), and a fiber collector composed of a rotating drum formed by 22-mm diameter nylon threads. FDA-approved PLA polymer (TM INGEO NatureWorks, 3251D, batch ZC2428B113, melting temperature of 188-210 °C, specific gravity 1.24, Tg of 55-65 °C) was provided by Promoplast (Mexico). The PLA polymer (1.2 g) was dissolved in chloroform (9 ml) at room temperature. Prior to the electrospinning process, 1 ml of ethanol was added to the solution. An electric field between the needle and the collector, located 5.5 cm apart from each other, was produced by applying a 20 kV voltage. The collection time was set to 1 h at a constant temperature of 26 ºC and a relative humidity of 58%, unless otherwise stated. Finally, electrospun scaffolds were maintained at 50 ºC for one week to remove the chlorine residues. 

**Plasma polymerization**

**Fig. 1:**
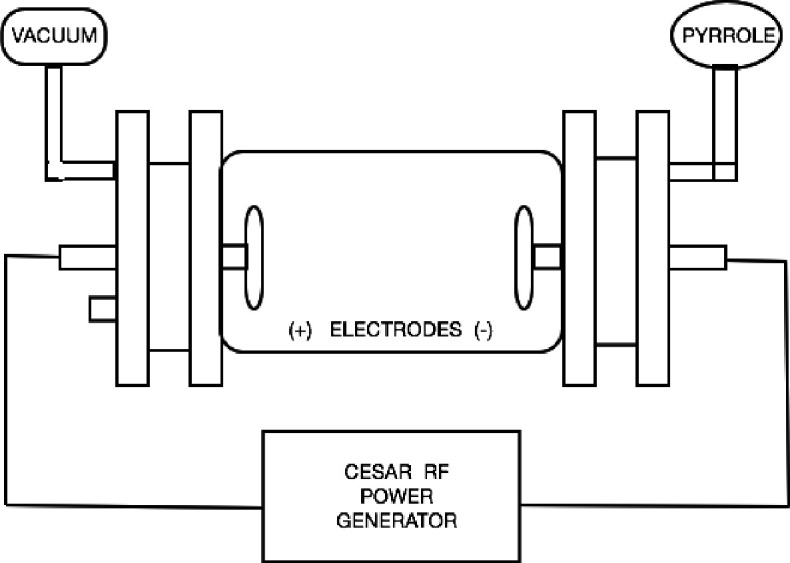
Configuration of plasma reactor used in the polymerization processes of the materials.

**Fig. 2:**
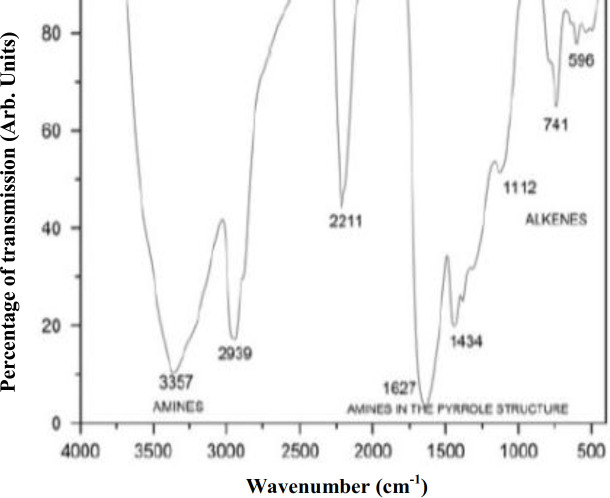
FT-IR of PPy film to know the functional groups present in the films.

Coverslips and electrospun PLA scaffolds were coated by plasma PPy synthesized in a low pressure glow discharge reactor using a pyrrole monomer (98%; Sigma-Aldrich, USA). The reactor used (shown schematically in Fig. 1) consisted of a tubular glass with two stainless steel electrodes (active and grounded). Both coverslips and electrospun scaffolds were placed in the plasma reactor for 10 min at an oscillation frequency of 13.5 MHz using a power of 30 W (Dressler Cesar, RF Power Generator). To ensure sterility, the coated coverslips and scaffolds were placed under a UV light by 4 h. Functional groups of the polymerized coverslips, and scaffolds were identified using FT-IR (Perkin Elmer, USA). For this purpose, a potassium bromide (KBr) disk was placed in the plasma reactor during the polymerization process. Once polymerized, the KBr disk was analyzed by FT-IR at a pressure of 9 tons for 5 min. The FT-IR spectrum of PPy is shown in Figure 2. The characteristics peaks corresponding to the functional groups of PPy were observed^[19,21]^.

**Spinal cord dissection**

Mouse embryonic motoneurons were obtained from a pregnant female mouse bred at the bioterium of the Universidad Autonoma Metropolitana (Mexico) as previously described^ [27]^. The mouse was sacrificed by inhalation of CO_2_. Then the embryos were carefully removed and placed on a Petri dish containing HBSS preheated at 21 ºC. With a pair of tweezers, both the head and tail of the embryo were removed. With the embryo back side, a pair of tweezers was used to remove the thin outer layer of skin while using the other pairs to hold the embryo. The central channel of the lumbar spinal cord was opened by sliding a pair of tweezers along the midline. The lumbar region was obtained by cutting the spinal cord along the L1-L6 segment. The tweezers were then inserted to lift the spinal cord up with saw-like movements on both sides. The lumbar region was then placed in a new dish containing HSBB, placing the open central channel side downwards to remove the dorsal root ganglia by displacing the encapsulant meninges. The L1-L6 segment of the spinal cord was used for the subsequent procedures. Depending on the gestational status, the spinal cord contained between 5,000 and 10,000 embryo motoneurons^[27]^. Each spinal cord was dissected and stored in a 1.5-ml microcentrifuge tube with 180 µl of HBSS and subsequently placed on ice. Trypsin 0.25% (10 µl) was applied for 20 min, stopping the reaction using neurobasal medium (1 ml) with 10% fetal bovine serum (all obtained from Gibco, USA). The tissue was first gently triturated 20 times using 1-ml pipette tips and was triturated again 20 times using 200-µl pipette tips. 

**Purification of motoneurons**

Neurons were purified in a 100-mm culture dish previously coated with lectin at a concentration of 10 µg/ml. The lectin used was prepared in 10 mM of TRIS solution (J.T. BAKER, USA). Then the dishes were covered with lectin (10 mL) and placed in an incubator for 30 min. Dishes were washed three times with HBSS and left covered with HBSS until use. Cells were placed in a lectin-covered culture dish (filled with HBSS) where they were homogenized by pipetting and by gently shaking the culture dish. While keeping covered to avoid contamination, cells were allowed to attach to the lectin-coated culture dish at room temperature for 60 min. Cell fragments were removed by softly washing with HBSS preheated at 23ºC. Immediately after the last wash, depolarization solution (500 µl, 30 mM of KCl, and J.T. Baker 0.9% w/v NaCl, JT. Baker) was added to the culture dish, which was then placed in an incubator for 1 min. The depolarization solution was removed, and the cells were finally washed with HBBS. Purified cells were quantified after the whole process, yielding a final count of 300 × 10^3^ cells.

**Cultures of motoneurons on electrospun PLA and PPy-coated PLA fibers**

To verify that the purified motoneurons were not damaged by the purification or depolarization processes, we analyzed the morphological, electro-physiological, and biochemical characteristics of motoneurons cultured on glass coverslips coated with Poly-DL-Ornitine/laminin and glass coverslips coated with PPy. Once proper cellular behavior was confirmed, purified motoneurons were cultured on PLA and PPy-coated PLA fibers. Motoneurons were then placed in a 15 ml tube and counted in a Neubauer chamber. About 100 × 10^3^ cells were seeded in 1 cm^2^ of PLA and PPy-coated PLA fibrous scaffolds and suspended in culture medium. Once proper anchorage was observed, the medium was replaced with an enriched medium to enhance the growth and differentiation of the motoneurons^[19,27]^.

**Fig. 3:**
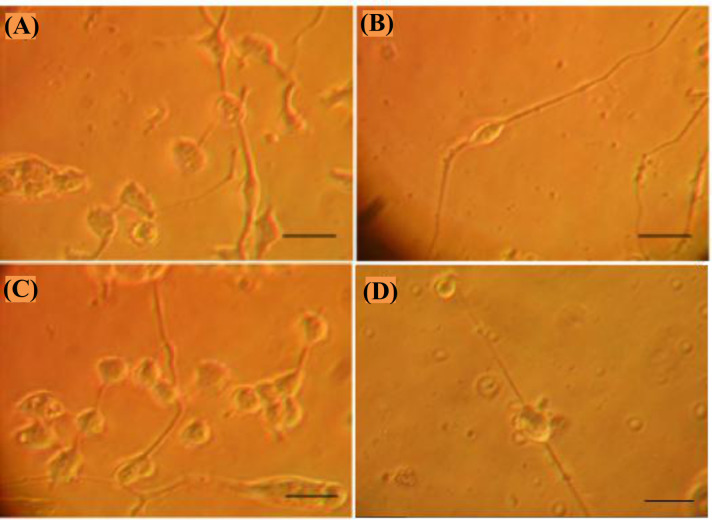
Purified motoneurons cultured in Poly-DL-Ornithine/Laminin at days (A) 3 and (B) 7 of culture. Purified motoneurons cultures in PPy-coated coverslips at days (C) 3 and (D) 7 of culture (phase contrast, bar = 50 microns; original magnification ×100).

**Labeling the VAChaT using immunofluorescence techniques**

Cells were fixed with 4% paraformaldehyde and 25% glutaraldehyde for 10 min. Cell membranes were permeabilized with 0.1% Triton (Sigma-Aldrich) for 10 min. Thereafter, the samples were washed five times with PBS. Motoneurons were labeled using the antibody anti-VAChaT (1:800) in conjunction with FITC (1:400). Cells were observed through a confocal microscope (LSM 710 AxioObserver ,Carl Zeiss SMT, Inc., Oberkochen, Germany). Cell nuclei were labeled with Hoeschst (1:500), and the cells were mounted using fluoroshield.

**Whole-cell voltage and current-clamp recordings**

Whole-cell voltage and current-clamp recordings were performed using an Axopatch 200A amplifier (Molecular Devices, CA, USA). For the voltage-clamp experiments, test pulses from -60 to 30 mV were imposed from a holding potential of -70 mV; under these conditions, current rectangular pulses for the measurement of the passive properties of cell membranes and inducing action potentials were applied. Stimulation protocols (both in voltage- and current-clamp modes) and voltage and current responses were digitized with an analog-digital board (Digidata1200, Molecular Devices, CA, USA). Recorded signals were analyzed using Clampfit 10.2 (Molecular Devices, CA, USA) and GraphPad Prism 5.0 (Graphpad Software Inc., CA, USA). Electrophysiological recordings were carried out at room temperature. Whole-cell currents and action potentials were recorded in an extracellular solution containing (in mM) 4.5 of KCl, 151.5 of NaCl, 1 of MgCl_2_, 2 of CaCL_2_, and 5 of HEPES (J.T. Baker). Electrodes were pulled from borosilicate glass capillaries (World Precision Instruments, FIL glass fIN 1BBL NO, USA) using a horizontal puller (Sutter Instruments, Model P-97) to obtain a resistance of ~2.4 MOhms. Pipettes were filled with an intracellular solution containing (in mM) 140 of KCl, 10 of NaCl, 1 of MgCl_2_, 1 of CaCl_2_, 11 of EGTA, and 10 of HEPES (J.T. Baker).

## RESULTS

**Culture of motoneurons on poly-DL-Ornithine/ Laminin- and PPy-coated coverslips**

In Figure 3A, motoneurons cultured on Poly-DL-Ornithine/Laminin are shown at day three of culture. Motoneurons had a body diameter of 20-30 µm in the range of the characteristic diameters reported in the literature^[19,27,28]^. The development of neural processes was observed during the first days of culture. As can be observed in Figure 3B, there are two long neural processes arising from the soma of a bipolar neuron at day seven of culture, resembling the characteristic morphology previously reported^[19,27,28]^. As represented in Figure 3C, motoneurons cultured on PPy surfaces at day three of culture showed a similar morphology to those cultured on Poly-DL-Ornithine/laminin, including the cell body diameter between 20 and 30 µm and the development of neural processes. At day seven of culture (Fig. 3D), motoneurons completed their development and showed long neural processes as reported before^[19]^. 

**Fig. 4:**
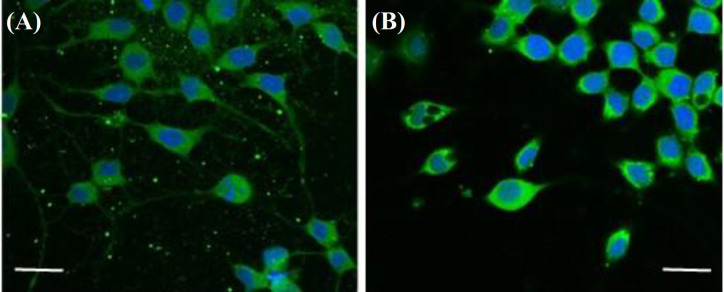
Fluorescence microscopy of purified motoneurons at day 3 of culture in (A) Poly-DL-Ornithine/Laminin and (B) PPy-coated coverslips (FITC-Anti-VChaT: green; Hoechst: blue; bar = 50 microns; original magnification ×100).

**Immunofluorescence on 2D cultures**

At day five of culture, we evaluated the presence of VAChaT by staining with an anti-VAChT antibody in conjunction with a FITC fluorochrome. As depicted in Figure 4, the presence of VAChaTs was successfully identified in the motoneurons cultured in both Poly-DL-Ornithine/Laminin and PPy, which demonstrated that the surface coating with PPy does not affect the growth of motoneurons.

**Electrophysiological recordings on 2D cultures**

**Fig. 5:**
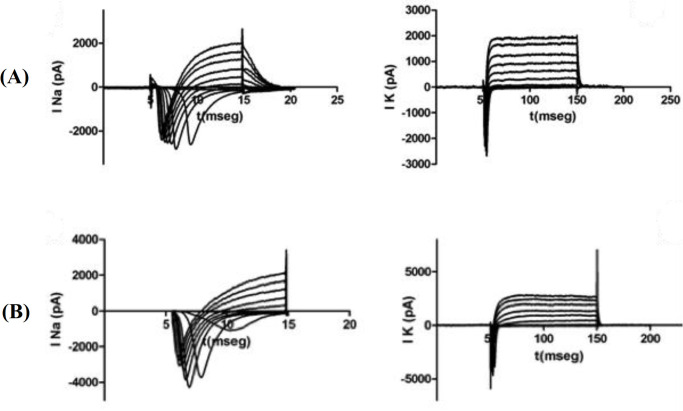
**(**A) Whole-cell Na^+^ and K^+^ currents recorded from motoneurons cultured on Poly- DL-Ornithine/Laminin-coated coverslips; (B) hole-cell Na^+^ and K^+^ currents recorded from motoneurons cultured on PPy-coated coverslips (representative of n cases).

Whole-cell Na^+^ and K^+^ currents were recorded in both preparations at day five of culture. Na^+^ currents were elicited by short depolarizing voltage pulses (10 ms), as shown in Figure 5, corresponding to motoneurons cultured on surfaces coated with Poly- DL- Ornithine/Laminin and PPy. In both cases, Na^+^ currents indicated a transient behavior, reflecting the fast activation and inactivation processes. Similarly, in Figures 5, we showed the temporal course of the K^+^ currents through the K^+^ delayed rectifying channels, recorded from motoneurons at day five of culture on culture on surfaces coated with Poly-DL- Ornithine/ Laminin and PPy, respectively, in response to 100 ms depolarizing voltage pulses. As expected, K^+^ currents demonstrated a slower activation process with no apparent inactivation during sustained depolarization. 

**Fig. 6:**
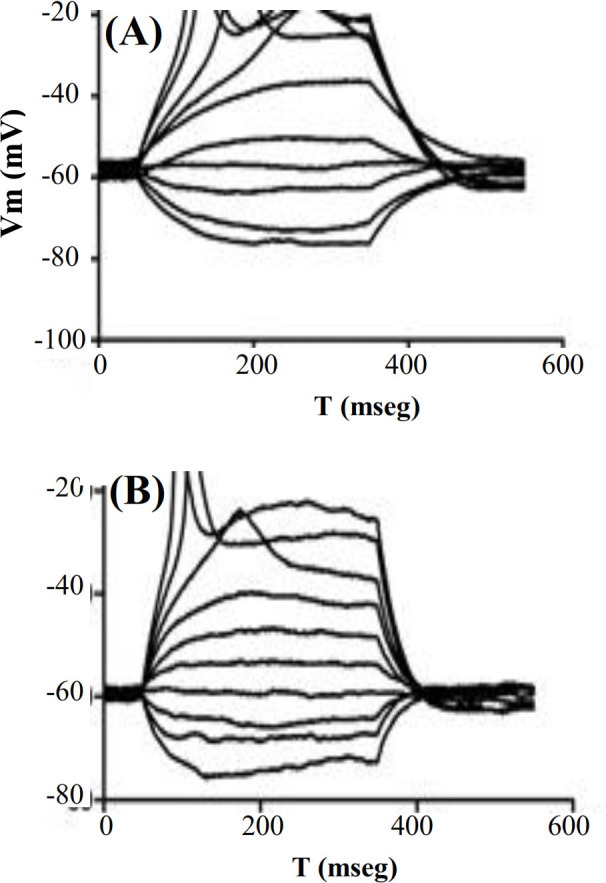
Action potentials in motoneurons cultured on (A) Poly-DL-Ornithine/Laminin- and (B) PPy-coated coverslips (representative of n cases).

Action potentials produced in response to current stimulation in motoneurons at day five of culture on surfaces coated with Poly-DL-Ornithine/Laminin, and PPy is shown in Figure 6. In both cases, the action potentials reached a peak voltage value around -10 mV.

**Culture of motoneurons on electrospun PLA and PPy-coated PLA fibers**

**Fig. 7:**
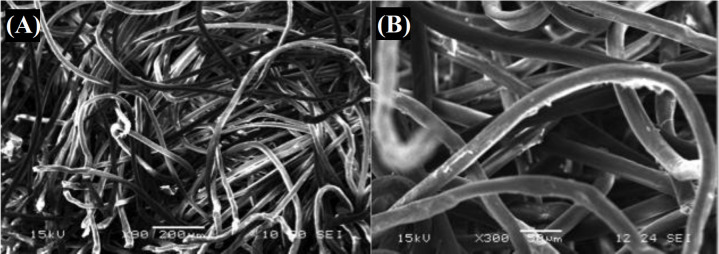
(A) SEM images of the internal structure of electrospun PLA fibers (bar = 200 microns; original magnification ×90); (B)PPy-coated PLA fibers (bar = 50 microns; original magnification ×300)

**Fig. 8:**
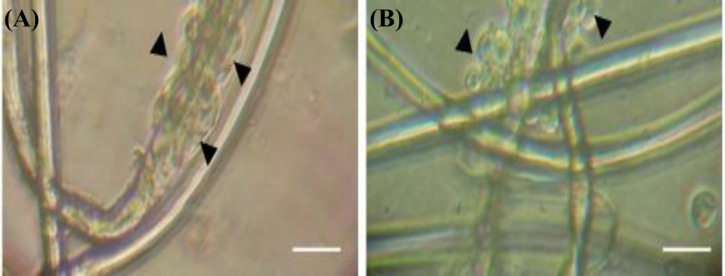
Motoneurons seeded in (A) PLA electrospun fibers and (B) PPy-coated PLA. Arowheads indicate the location of motoneurons (phase contrast; bar = 50 microns; original magnification ×200).

A SEM image of the internal structure of uncoated PLA electrospun fibers is shown in Figure 7A. Uncoated PLA fibers had a diameter ranging between 20 and 25 µm. The same range of diameters was observed in PPy-coated fibers, as can be observed in Figure 7B. Motoneurons cultured on PLA and PPy-coated PLA fibrous scaffolds (at day five of culture) are shown in Figure 8A and 8B, respectively. In both cases, motoneurons showed a spherical morphology and formed clusters around the fibers. Interestingly, motoneurons did not develop neural processes in neither of the two scaffolds used, probably due to the limited surface available for the motoneurons to attach to the fibers.

**Immunofluorescence**

**Fig. 9:**
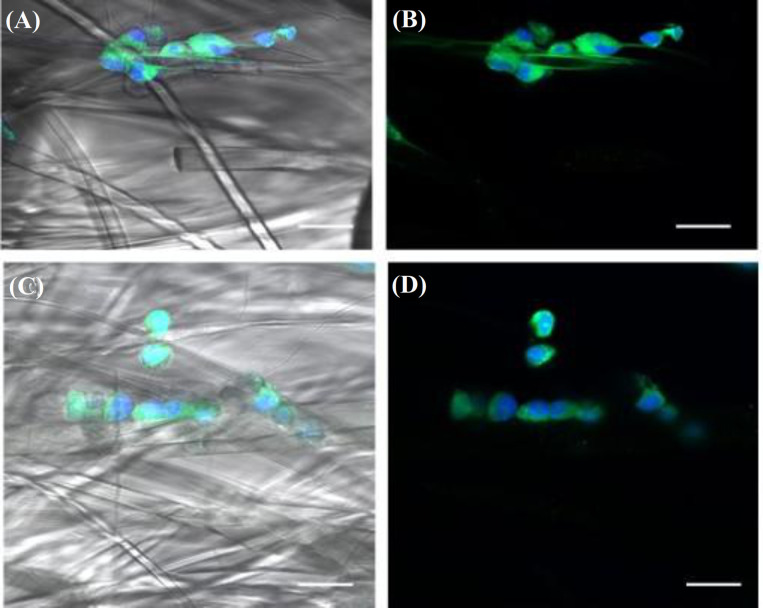
Motoneurons cultured on (A and B) PLA fibers and (C and D) PPy-coated PLA fibers (Anti-VChaT-FITC: green; Hoechst: blue; bar = 50 microns; original magnification ×400)

In Figure 9, the fluorescence images of motoneurons at day five of culture on PLA (A and B) and PPy-coated PLA (C and D) scaffolds are illustrated. The immunofluorescence assay confirmed the observation that motoneurons cultured in electrospun scaffolds showed a clustered growth, adopted a spherical morphology, and did not develop noticeable neural processes.

**Electrophysiological recordings on 3D cultures**

**Fig. 10:**
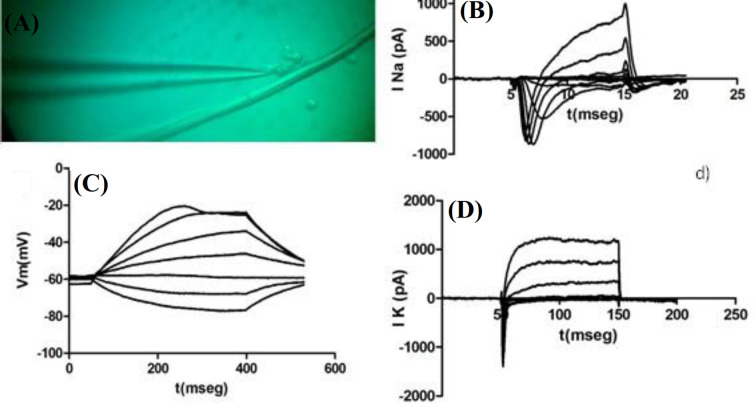
(A) Record cell photomicrograph, (B) Na^+^ ionic currents, (C) action potential, and (D) K^+^ ionic currents of motoneurons seeded on PLA fibers (representative of n cases).

**Fig. 11:**
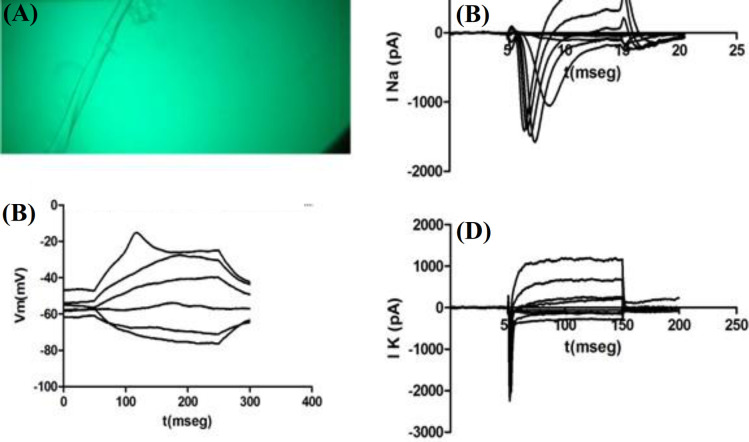
(A) Record cell photomicrograph, (B) Na^+^ ionic currents, (C) action potential, and (D) K^+^ ionic currents of motoneurons seeded on PLA-PPy fibers (representative of n cases).

Whole-cell Na^+^ and K^+^ currents and also action potentials were recorded in motoneurons cultured on PLA and PPy-coated PLA scaffolds (at day five of culture), as shown in Figures 10A and 11A, respectively, where the recorded cells can be found along with the recording microelectrode. Based on Figures 10B and 11B, Na^+^ currents recorded from cultured motoneurons on both types of fibrous scaffolds showed a lower amplitude if compared to the Na^+^ currents recorded from motoneurons cultured on either of the coated coverslips. Similarly, K^+^ currents depicted in Figures 10D and 11D, corresponding to the motoneurons cultured on PLA and PPy-coated PLA scaffolds, also exhibited a lower amplitude than the K^+^ current recorded from motoneurons cultured on the coated coverslips. The action potentials recorded from motoneurons cultured on the fibrous scaffolds differed considerably with respect to those recorded from motoneurons cultured on the coated coverslips. In particular, motoneurons cultured on both PLA and PPy-coated PLA scaffolds were only capable of producing a slow action potential in response to current stimulation when compared to those produced by the motoneurons cultured on the coated coverslips (Figs. 10C and 11C,). In addition, action potentials showed a lower amplitude around -20 mV than those recorded from motoneurons cultured on the coated coverslips.

## DISCUSSION

Plasma polymerization allowed us to obtain a thin layer of PPy on the surface of electrospun PLA fibers, which were evaluated as growth surfaces for purified mouse embryo motoneurons. Both PLA and PPy-coated PLA fibrous scaffolds promoted the attachment of motoneurons, as observed through optical microscopy and immunofluorescence assays, while simultaneously allowing the proper nutrition of the cells by permitting the free passage of nutrients through the intrafiber pores. However, motoneurons cultured on the fibrous scaffolds were not capable of developing the neural processes commonly observed in motoneurons cultured on 2D surfaces. We hypothesize that the limited growth of neural processes 

In PPy-coated PLA fibrous scaffolds motoneurons cultured on fibrous scaffolds is due to the limited surface available for the motoneurons to attach to the fibers, although it is also possible that neural processes developed along the fibers were not visible through conventional or confocal microscopy. Given that practically the same morphological characteristics were detected in motoneurons cultured on coverslips coated with Poly-DL-Ornithine/laminin and PPy, we can conclude that neither substrate affected the morphological development of the motoneurons. In spite of this observation, we were able to confirm the growth of motoneurons in the fibrous scaffolds by VAChT labeling.

Electrophysiological recordings of the whole-cell Na^+^ and K^+^ currents produced by motoneurons cultured on PLA and PPy-coated PLA scaffolds showed that motoneurons cultured on the fibrous scaffolds produce Na^+^ and K^+^ currents with a lower amplitude when compared to the same currents recorded from the motoneurons cultured on coated coverslips. Similarly, action potentials observed in the cells cultured on the fibrous scaffolds suggested both a slower temporal course and a lower amplitude if compared to the action potentials recorded from motoneurons cultured on the PPy-coated PLA fibrous scaffolds. We suppose that the lower amplitude of both the Na^+^ and K^+^ currents, as well as the action potentials obtained from the motoneurons cultured on the 3D fibrous scaffolds could be produced by the limited growth of neural processes. These results indicate that PPy as a growth surface does not affect the firing of action potentials. Given that the whole-cell Na^+^ and K^+^ currents as well as the firing of action potentials were not significantly affected by PPy (when compared to the response obtained from motoneurons cultured on Poly-DL-Ornithine/laminin), it can be concluded that PPy does not influence the electrophysiological properties of the motoneurons.

In this work, we used a lectin-based purification process to obtain mouse embryo motoneurons with proper electrical activity, adequate cell morphology, and the presence of acetylcholine VAChT transporters, using coverslips coated with both Poly-DL-Ornithine/laminin and PPy as culture surfaces. A 3D model was then developed by culturing the motoneurons on PLA and PPy-coated PLA electrospun fibers. The cultured motoneurons showed a satisfactory attachment and also a limited morphological development, which could be due to the limited growth area available in the micrometric fibers. We were able to confirm the presence of VAChaTs both in the coated coverslips and in the 3D cultures. In addition, in all cases, cultured motoneurons produced action potentials as well as Na^+^ and K^+^ whole-cell currents. However, it should be noted that the currents and action potentials produced by the cells cultured on the fibrous scaffolds displayed lower amplitude when compared to those cultured on the coated coverslips, which could be an undesired effect of the purification process. These results suggest that the cultures of motoneurons on fibrous scaffolds not only affects the development of neural process in mouse motoneurons but also influences the expression of ionic channels and/or their functional properties, which is reflected on their electrophysiological response.

According to our results, electrospun fibers and the purification process might influence the normal development of the cultured motoneuron. The method of purification motor neurons with glial cell-free may affect the proper growth and functional development of the motor neuron. This would seem to be the case, due to the short half-life (5-7 days) that has been reported in two-dimensional cultures. Likewise, we know that glial cells are fundamental for neurons; it is possible that their absence limits their growth and functional development, and this is aggravated in 3D cultures. Therefore, we do not suggest the use of purified motor neurons in the study of biomaterials for 3D cultures.

## DECLARATIONS

### Acknowledgments

Authors would like to acknowledge Dr. Rafael Godinez-Fernandez, Dr. Roberto Olayo Gonzalez, and Dr. Juan Morales Corona for preparation of the materials and technical support during the experimentation time in the UAM-Iztapalapa Laboratories.

### Ethical statement

The above-mentioned sampling protocols were approved by the Ethical Board for Animal Experiments of the Universidad Autonoma Metropolitana, in compliance with the guidelines of the National Bioethics Commission and NOM-062-ZOO-1999 (ConBioética, Mexico; ethical code: NOM-062-ZOO-1999). 

### Data availability

The raw data supporting the conclusions of this article are available from the authors upon reasonable request. The analyzed data sets generated during the study are available from the corresponding author on reasonable request.

### Author contributions

EZ: experimental procedures, data analysis, writing, and editing; OR: experimental procedures, editing; CM: writing and editing.

### Conflict of interest

None declared.

### Funding/support

This project did not receive funding.

## References

[1] Qutub A, Popel A (2009). Elongation, proliferation and migration differentiate endothelial cell phenotypes and determine capillary sprouting. BMC systems biology.

[2] Ayala P, Lopez J, Desai T (2010). Microtopographical cues in 3D attenuate fibrotic phenotype and extracellular matrix deposition: implications for tissue regeneration. Tissue engineering. Part A.

[3] Bott K, Upton Z, Schrobback K, Ehrbar M, Hubbell J, Lutolf M (2010). The effect of matrix characteristics on fibroblast proliferation in 3D gels. Biomaterials.

[4] Li C, Tian T, Nan K, Zhao N, Guo Y, Cui J (2008). Survival advantages of multicellular spheroids vs monolayers of HepG2 cells in vitro. Oncology reports.

[5] Myers T, Nickerson C, Kaushal D, Otte C, Bentrup K, Ramamurthy R, Nelman M, Pierson D, Philipp M (2008). Closing the phenotypic gap between transformed neuronal cell lines in culture and untransformed neurons. Journal of neuroscience methods.

[6] Irons H, Cullen D, Shapiro N, Lambert N, Lee R, LaPlaca M (2008). Three-dimensional neural constructs: a novel platform for neurophysiological investigation. Journal of neural engineering.

[7] Yang F, Murugan R, Wang S, Ramakrishn S (2005). Electrospinning of nano/micro scale poly(L- lactic acid) aligned fibers and their potential in neural tissue engineering. Biomaterials.

[8] Corey JM, Gertz CC, Bor-Shuen W, Birrell LK, Johnson SL, Martin DC, Feldman LF (2008). The design of electrospun PLLA nanofiber scaffolds compatible with serum-free growth of primary motor and sensory neurons. Acta biomaterialia.

[9] Fabela-Sánchez O, Salgado–Ceballos H, Medina-Torres L, Álvarez-Mejía L, Sánchez- Torres S, Mondragon-Lozano R, Morales-Guadarrama A, Diaz-Ruiz A, Olayo MG, Guillermo J, Cruz G, Morales-Corona J, Ríos C, Olayo R (2018). Effect of the combined treatment of albumin with plasma synthesised pyrrole polymers on motor recovery after traumatic spinal cord injury in rats. Journal of materials science materials in medicine.

[10] Lee J, Cuddihy M, Kotov N (2008). Three-Dimensional Cell Culture Matrices: State of the Art. Tissue engineering part B: reviews.

[11] Cukierman E, Pankov R, Yamada KM (2002). Cell interactions with three-dimensional matrices. Current opinion in cell biology.

[12] Xie J, MacEwan M, Schwartz A, Xia Y (2010). Electrospun nanofibers for neural tissue engineering. Nanoscale.

[13] Cao H, Liu T, Chew S (2009). The application of nanofibrous scaffolds in neural tissue engineering. Advanced drug delivery reviews.

[14] Subramanian A, Maheswari KU, Sethuraman S (2009). Development of biomaterial scaffold for nerve tissue engineering: Biomaterial mediated neural regeneration. Journal of biomedical science.

[15] Yao L, O'Brien N, Windebank A, Pandit A (2009). Orienting neurite growth in electrospun fibrous neural conduits. Journal of biomedical materials research part B: applied biomaterials.

[16] Olayo R, Ríos C, Salgado CH, Morales J (2008). Tissue spinal cord response in rats after implants of polypirrole and polyethylene glycol obtained by plasma. Journal of materials science: materials in medicine.

[17] Zuñiga-Aguilar E, Godinez R, Morales MA, Cifuentes F, Ramírez-Fernández O, Morales J, Olayo R (2013). Crecimiento de células nerviosas motoras sobre material modificado superficialmente por polimerización por plasma. IFMBE proceedings.

[18] Zuñiga-Aguilar E, Olayo R, Ramírez-Fernández O, Morales J, Godínez R (2014). Nerve cells culture from lumbar spinal cord on surfaces modified by plasma pyrrole polymerization. Journal of biomaterials science. Polymer edition.

[19] Pérez-Tejada E, Morales-Corona J, Gómez-Quiróz LE, Gutierrez-Ruiz MC, Olayo R (2018). Effect of synthesis variables of plasma synthesized polymers on growth of HepG2 cells. Biocell.

[20] Ramírez – Fernández O, Godínez R, Morales J, Gómez-Quiroz L, Gutiérrez-Ruiz MC, Zuñiga-Aguilar E, Olayo R (2012). Superficies modificadas mediante polimerización por plasma para cocultivos de modelos hepáticos. Revista mexicana de ingeniería biomédica.

[21] Cruz JG, Mondragón-Lozano G, Díaz-Ruiz A, Manjarrez J, Olayo R, Salgado-Ceballos H, Olayo MG, Morales J, Alvarez-Mejía L, Morales A, Méndez-Armenta M, Plascencia N, Fernández M, Ríos C (2012). Plasma polypyrrole implants recover motor function in rats after spinal cord transection. Journal of materials science: materials in medicine.

[22] Dhillon A, Kaur A, Srivastava AK, Avasthi DK (2010). Experimental investigations of semi-crystalline plasma polymerized polypyrrole for surface coating. Progress in organic coatings.

[23] Zhang J, Wu MZ, Pu TS, Zhang ZY, Jin RP, Tong ZS, Zhu DZ, Cao DX, Zhu FY, Cao JQ (1997). Investigation of the plasma polymer deposited from pyrrole. Thin solid films.

[24] Wang J, Neoh KG, Kang ET (2004). Comparative study of chemically synthesized and plasma polymerized pyrrole and thiophene thin films. Thin solid films.

[25] Serratos IN, Olayo R, Millán-Pacheco C, Morales-Corona J, Vicente-Escobar JO, Soto-Estrada AM, Godínez-Fernández R (2019). Modeling integrin and plasma-polymerized pyrrole interactions: chemical diversity relevance for cell regeneration. Scientific reports.

[26] Wichterle H, Lieberam I, Porter J, Jessel T (2002). Directed differentiation of embryonic stem cells into motor neurons. Cell.

[27] Wiese S, Herrmann T, Drepper C, Jablonka S, Funk N, Klausmeyer A, Rogers ML, Rush R, Sendtner M (2010). Isolation and enrichment of embryonic mouse motoneurons from the lumbar spinal cord of individual mouse embryos. Nature protocols.

[28] Hubbard KS, Gut IM, Scheeler SM, Lyman ME, McNutt PM (2012). Compatibility of SYTO 13 and Hoechst 33342 for longitudinal imaging of neuron viability and cell death. BMC research notes.

